# The clinical value of metabolic syndrome and risks of cardiometabolic events and mortality in the elderly: the Rotterdam study

**DOI:** 10.1186/s12933-016-0387-4

**Published:** 2016-04-27

**Authors:** Thijs T. W. van Herpt, Abbas Dehghan, Mandy van Hoek, M. Arfan Ikram, Albert Hofman, Eric J. G. Sijbrands, Oscar H. Franco

**Affiliations:** Department of Epidemiology, Erasmus Medical Center Rotterdam, PO Box 2040, 3000 CA Rotterdam, The Netherlands; Department of Internal Medicine, Erasmus Medical Center Rotterdam, PO Box 2040, 3000 CA Rotterdam, The Netherlands; Department of Neurology, Erasmus Medical Center Rotterdam, PO Box 2040, 3000 CA Rotterdam, The Netherlands; Department of Radiology, Erasmus Medical Center Rotterdam, PO Box 2040, 3000 CA Rotterdam, The Netherlands

**Keywords:** Metabolic syndrome, Cardiovascular disease, Diabetes mellitus, Stroke, Cardiovascular mortality, All-cause mortality, Preventive medicine, Rotterdam study

## Abstract

**Background:**

To evaluate the clinical value of metabolic syndrome based on different definitions [American Heart Association/National Heart, Lung and Blood Institute (AHA/NHLBI), International Diabetes Federation (IDF) and European Group for the Study of Insulin Resistance (EGIR)] in middle-aged and elderly populations.

**Methods:**

We studied 8643 participants from the Rotterdam study (1990–2012; mean age 62.7; 57.6 % female), a large prospective population-based study with predominantly elderly participants. We performed cox-proportional hazards models for different definitions, triads within definitions and each separate component for the risk of incident type 2 diabetes mellitus, coronary heart disease, stroke, cardiovascular- and all-cause mortality.

**Results:**

In our population of 8643 subjects, metabolic syndrome was highly prevalent (prevalence between 19.4 and 42.4 %). Metabolic syndrome in general was associated with incident type 2 diabetes mellitus (median follow-up of 6.8 years, hazard ratios 3.13–3.78). The associations with coronary heart disease (median follow-up of 7.2 years, hazard ratios 1.08–1.32), stroke (median follow-up of 7.7 years, hazard ratios 0.98–1.32), cardiovascular mortality (median follow-up of 8.2 years, ratios 0.95–1.29) and all-cause mortality (median follow-up of 8.7 years, hazard ratios 1.05–1.10) were weaker. AHA/NHLBI- and IDF-definitions showed similar associations with clinical endpoints compared to the EGIR, which was only significantly associated with incident type 2 diabetes mellitus. All significant associations disappeared after correcting metabolic syndrome for its individual components.

**Conclusions:**

Large variability exists between and within definitions of the metabolic syndrome with respect to risk of clinical events and mortality. In a relatively old population the metabolic syndrome did not show an additional predictive value on top of its individual components. So, besides as a manner of easy identification of high risk patients, the metabolic syndrome does not seem to add any predictive value for clinical practice.

**Electronic supplementary material:**

The online version of this article (doi:10.1186/s12933-016-0387-4) contains supplementary material, which is available to authorized users.

## Background

The metabolic syndrome (MetS) is a combination of risk factors for type 2 diabetes mellitus and cardiovascular disease (CVD). Although MetS was designed to cluster and predict risk for type 2 diabetes mellitus and CVD, controversy remains on its usefulness in clinical practice. This is due to the fact that it is still not fully clear whether MetS has an added value to the prediction of diabetes, cardiovascular disease and mortality above the effect of its individual components [1–6].

There are a number of different definitions according to which MetS can be defined which may have led to heterogeneity. The currently applied definitions have substantial differences in the predefined components and cut-off values [7–10]. Furthermore, most studies on the association between MetS and cardiovascular disease, mortality and diabetes have been performed in middle-aged populations [11–15] while the associations of MetS with type 2 diabetes mellitus, CVD and mortality and the added value of MetS above its individual components in elderly populations has received less attention and has led to inconsistent results [2–5, 16–19].

Therefore the aim of our study was to determine the clinical value of MetS in a large prospective Dutch predominantly elderly population comparing three commonly applied definitions. We investigated the associations of the definitions, their exact composition and the added predictive value above their individual components with risk of type 2 diabetes mellitus, coronary heart disease (CHD), stroke, cardiovascular—and all-cause mortality.

## Methods

### Study population

Analyses were performed in the Rotterdam study, an ongoing prospective population-based cohort study in Rotterdam, The Netherlands. In 1989, all residents aged 55 years or older in a well-defined district of Rotterdam were invited to participate in the original cohort (RS-I). A total of 7983 (78.1 %) agreed to participate in the follow-up study. The study was extended in 2000 with a cohort of individuals who had reached age 55 or moved into the study area after the initial cohort (n = 3011). In 2006, a third cohort of 3932 participants aged 45 years or older was enrolled, bringing the total study size to 14,926 individuals. There were no eligibility criteria to enter the Rotterdam study cohorts except the minimum age and residential area. A more detailed description of the methods of the Rotterdam study can be found elsewhere [20, 21].

Participants are being monitored for type 2 diabetes mellitus, CHD, stroke and mortality by continuous linkage to files from general practitioners in the study area, information from medical specialists and discharge reports after hospitalization. All information was obtained through trained research employees and reviewed by two independent medical doctors, supervised by a specialist in each separate medical field.

The Rotterdam study has been approved by the medical ethics committee according to the population study act Rotterdam study, executed by the Ministry of Health, Welfare and Sports of the Netherlands. Written informed consent was obtained from all participants.

### Population for analysis

A total of 14,926 participants were included in three subsequent cohorts in the Rotterdam study. From the first cohort entering the study in 1990 (n = 7983) we used data from their third examination (n = 4797, 1997–1999) because of the availability of fasting blood samples. Furthermore we used data from participants of the second (n = 3011, 2000–2001) and third cohort (n = 3932, 2006–2008).

From the 11,740 participants mentioned above, 10,599 went to the research center for blood sampling and anthropometric measurements. Only fasting participants were included in the study (n = 9819) Subsequently, we excluded 1176 prevalent cases of type 2 diabetes mellitus resulting in a population for analysis of n = 8643. For each given endpoint, prevalent cases of that endpoint where excluded. An average of 1.7 % had missing data on MetS-components. These were imputed by using the multiple imputation method described by Sterne et al. [22].

### Definitions of MetS

MetS was defined according to 3 definitions (Additional file 1: Table S1): (1) as stated by the American Heart Association/National Heart, Lung and Blood Institute (AHA/NHLBI) [8], which was later used without adjustments in the consensus definition of IDF and AHA/NHLBI in 2009 [10], (2) according to the International Diabetes Federation (IDF) [9] and (3) according to the European Group for the Study of Insulin Resistance (EGIR) [7]. A diagnosis of MetS according to AHA/NHLBI-criteria consists of at least 3 of the following components: (1) waist circumference >102 cm for males or >88 cm for females; (2) HDL-cholesterol <1.03 mmol/l for males or HDL-cholesterol <1.29 mmol/l for females, (3) triglycerides ≥1.7 mmol/l, (4) systolic blood pressure ≥130 mmHg or diastolic blood pressure ≥85 mmHg or antihypertensive treatment; and (5) fasting glucose of ≥5.6 mmol/l or drug treatment for elevated glucose.

According to IDF-criteria, a diagnosis of MetS includes the component of central obesity (COB) as defined by waist circumference ≥94 cm for males or waist circumference ≥80 cm for females. If BMI is >30 kg/m^2^, central obesity is assumed and waist circumference does not need to be measured. Central obesity is the central component in the definition of MetS according to IDF. In addition to central obesity, two of the following four components should be present: (1) raised triglycerides ≥1.7 mmol/l, (2) HDL-cholesterol <1.03 mmol/l for males or HDL-cholesterol <1.29 mmol/l for females, (3) systolic blood pressure ≥130 mmHg or diastolic blood pressure ≥85 mmHg or treatment of hypertension, (4) raised fasting plasma glucose ≥5.6 mmol/l or previously diagnosed type 2 diabetes mellitus. According to EGIR-criteria, the upper quartile of fasting insulin in a non-diabetes population is required together with two of the following components: (1) hyperglycemia ≥6.1 mmol/l but not having diabetes, (2) systolic blood pressure ≥140 mmHg or diastolic blood pressure ≥90 mmHg or treatment of hypertension, (3) dyslipidemia as defined by triglycerides >2.0 mmol/l or HDL-C <1.0 mmol/l, (4) central obesity as defined by a waist circumference ≥94 cm for males or waist circumference ≥80 cm for females.

### Components and triads

According to the AHA/NHLBI-, IDF- and EGIR-criteria we defined MetS at baseline. Triads where defined as the simultaneous combination within a participant of any three different components of the MetS that would guarantee a diagnosis of MetS (a participant could have >1 triad at the same time).

### Definition of type 2 diabetes mellitus

Incident type 2 diabetes mellitus was defined in accordance with the guidelines of the American Diabetes Association [23, 24] and World Health Organization (WHO) [25] as a (1) fasting glucose level ≥7.0 mmol/l or (2) a non-fasting glucose level ≥11.1 mmol/l or (3) treatment with oral glucose-lowering medication or insulin, and (4) diagnosis of diabetes as registered by a general practitioner or medical specialist. Prevalent cases of diabetes were diagnosed at baseline by a (1) non-fasting or post-load glucose level (after oral glucose tolerance test) ≥11.1 mmol/l or (2) treatment with oral glucose-lowering medication or insulin, and (3) diagnosis as registered by a general practitioner.

### Definition of CHD

Incident CHD was defined as (1) myocardial revascularization (as a proxy for significant coronary artery disease), (2) Myocardial Infarction (MI, fatal and nonfatal) and (3) fatal CHD. Specific details on definitions in each categories in the Rotterdam study can be found elsewhere [26].

### Definition of stroke

Stroke was defined according to WHO-criteria as a syndrome of rapidly developing clinical signs of focal (or global) disturbance of cerebral function, with symptoms lasting 24 h or longer or leading to death, with no apparent cause other than of vascular origin [27]. History of stroke at baseline was assessed during the baseline interview and verified by review of medical records. A more profound description on methods of data collection for stroke can be found elsewhere [28].

### Definition of cardiovascular mortality and all-cause mortality

Cardiovascular mortality was classified as mortality as a consequence of (1) CHD, (2) cerebrovascular disease, (3) atherosclerotic disease other than CHD or cerebrovascular disease (including ruptured abdominal aortic aneurysm, peripheral vascular disease, and visceral vascular disease) and (4) other cardiovascular disease. Specific details on definitions in each categories and methods of data collection of cardiac outcomes in the Rotterdam study can be found elsewhere [26]. With respect to all-cause mortality, information was obtained on a weekly basis from the central registry of the municipality in Rotterdam and through general practitioners working in the study area.

### Statistical analysis

Normally distributed continuous variables were expressed as mean ± standard deviation (SD). Continuous variables that were not normally distributed were log-transformed for the analysis and are expressed as a median with interquartile range. Age- and sex-adjusted logistic regression and Chi square tests were used to compare baseline characteristics of MetS and non-MetS participants. Cox proportional hazards models corrected for age, sex and ethnicity served to analyze the associated hazard ratio of MetS and incident type 2 diabetes mellitus, CHD, stroke, cardiovascular- and all-cause mortality. All models were initially adjusted for age, sex and ethnicity. Ethnicity did not have a significant effect in any of the models and was therefore left out. To investigate whether the metabolic syndrome as a syndrome captures more of the risk for clinical endpoints than the individual components, we subsequently corrected the hazard ratios of MetS for each individual component. We imputed missing values by using the multiple imputation method, which has been proven to be a reliable method [22]. Participants with prevalent or unknown disease status were excluded from analyses on type 2 diabetes mellitus, CHD and stroke. Participants with prevalent or unknown stroke and/or CHD status were excluded from the analyses on cardiovascular mortality. For the analysis on incident diabetes we performed sensitivity analyses in which we excluded participants with impaired fasting glucose levels (fasting glucose ≥5.6 mmol/l). All analyses were adjusted for age, sex and ethnicity. All analyses were performed with SPSS version 21.0 (SPSS, Chicago, IL, USA) and a 2-sided α smaller than 0.05 was used to claim statistical significance.

## Results

### Baseline characteristics

Baseline characteristics are shown in Table 1. The overall mean age at baseline was 62.7 years. Participants were more often female (57.6 vs. 42.4 %). Between definitions, the mean age of the participants having MetS ranged from 64.2 years (AHA/NHLBI) to 62.1 years (EGIR). From our study population, 97.8 % were of Caucasian descent. Other baseline characteristics are being displayed in Table 1.

**Table 1:** Baseline characteristics of population diagnosed with MetS according to different definitions

Characteristic	AHA/NHLBI	IDF	EGIR	Total population
Participants having MetS (n, %)	3055 (35.3)	3646 (42.2)	1680 (19.4)	8643
Age, years	64.2 (58.8–72.5)*	64.0 (58.8–72.6)*	62.1 (57.1–70.8)	62.7 (57.6–71.2)
Female sex (n, %)	1790 (58.6)	2090 (57.3)	919 (54.7)*	4983 (57.7)
Body mass index (kg/m^2^)	29.3 ± 4.1*	28.9 ± 3.9*	30.3 ± 4.3*	27.0 ± 4.1
Waist-circumference, cm	100.0 ± 10.8*	98.9 ± 10.5*	102.0 ± 11.2*	92.8 ± 11.8
Systolic blood pressure, mmHg	145.6 ± 19.0*	145.3 ± 19.1*	144.1 ± 18.9*	138.3 ± 20.8
Diastolic blood pressure, mmHg	81.4 ± 11.4*	81.3 ± 11.4*	82.6 ± 11.7*	78.8 ± 11.4
Total cholesterol, mmol/l	5.8 ± 1.1*	5.8 ± 1.1*	5.7 ± 1.1	5.8 ± 1.0
HDL cholesterol, mmol/l	1.2 (1.0–1.4)*	1.2 (1.0–1.4)*	1.2 (1.0–1.4)*	1.4 (1.1–1.7)
Triglycerides, mmol/l	1.8 (1.4–2.3)*	1.7 (1.3–2.2)*	1.8 (1.3–2.4)*	1.3 (1.0–1.8)
Insulin, pmol/L	92 (67–129)*	88 (64–123)*	131 (111–166)*	69 (49–97)
Glucose, mg/L	5.8 (5.4–6.1)*	5.7 (4.5–6.1)*	5.8 (5.4–6.2)*	5.4 (1.1–1.7)
CRP, mg/mL	2.2 (1.0–4.4)*	2.0 (0.9–4.1)*	2.2 (1.0–4.6)*	1.5 (0.6–3.3)
Hypertension treatment (n, %)	1045 (34.2)*	1197 (32.8)*	92 (41.2)*	1873 (21.7)
Antidiabetic treatment (n, %)	0 (0.0)	0 (0.0)	0 (0.0)	0 (0.0)
Lipid treatment (n, %)	563 (18.4)*	630 (17.3)*	353 (21.0)*	1255 (14.5)
Current smoking (n, %)	287 (9.4)	329 (9.0)*	114 (6.8)*	813 (9.4)
Caucasian descent (n, %)	2732 (98.2)	3255 (98.1)	1482 (96.9)	7655 (97.8)
Prevalent type 2 diabetes (n, %)	0 (0.0)	0 (0.0)	0 (0.0)	0 (0.0 %)
Prevalent CHD (n, %)	218 (7.1)*	249 (6.8)*	121 (7.2)*	518 (6.0)
Prevalent Stroke (n, %)	95 (3.1)*	107 (3.0)*	54 (3.2)*	196 (2.3)

Continuous data are mean ± SD when normally distributed. Otherwise median with interquartile range

*AHA/NHLBI*, American heart association/national heart, lung, and blood institute; *IDF*, International Diabetes Federation; *EGIR*, European Group for the Study of Insulin Resistance; *MetS*, metabolic syndrome; *HDL*, high-density lipoprotein; *CRP*, C-reactive protein; *CHD*, coronary heart disease

* Significant difference between MetS and non-MetS after correction for age and sex (P < 0.05)

### Prevalence of MetS

**Fig. 1:**
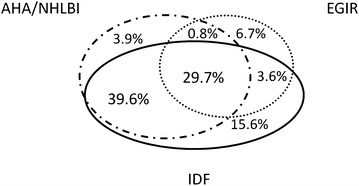
Concordance and disparity in diagnosis of metabolic syndrome according to different definitions

At baseline, a total of 4118 participants (47.6 %) were diagnosed with MetS according to either definition. The concordance of diagnoses using AHA/NHLBI, IDF and EGIR-definitions is displayed in Fig. 1. Thirty-five percent had a diagnosis according to AHA/NHLBI, 42.2 % according to IDF, and 19.4 % according to EGIR (Table 2).

**Table 2:** Prevalence of components/triads at RS-I-3 within diagnosis of MetS

	AHA/NHLBI	IDF	EGIR
Metabolic Syndrome	3055 (35.3 %)	3646 (42.2 %)	1680 (19.4 %)
Components within diagnosis	BP 2810 (92.0 %)	COB 3646 (100 %)	INS 1680 (100 %)
	WC 2319 (75.9 %)	BP 3302 (90.6 %)	WC 1627 (96.8 %)
	GLYC 2140 (70.0 %)	GLYC 2489 (68.3 %)	BP 1347 (80.2 %)
	HDL 1688 (55.3 %)	HDL 1750 (48.0 %)	DYSL 864 (51.4 %)
	TRIG 1849 (60.5 %)	TRIG 1896 (52.0 %)	GLYC 577 (34.3 %)
Triads within diagnosis	GLYC-BP-WC 1469 (48.1 %)	COB-GLYC-BP 2265 (62.1 %)	INS-WC-BP 1302 (77.5 %)
	BP-TRIG-WC 1075 (35.2 %)	COB-HDL-BP 1474 (40.4 %)	INS-WC-DYSL 821 (48.9 %)
	GLYC-BP-TRIG 1004 (32.9 %)	COB-TRIG-BP 1620 (44.4 %)	INS-BP-DYSL 611 (36.4 %)
	BP-HDL-WC 984 (32.2 %)	COB-TRIG-HDL 1074 (29.5 %)	INS-WC-GLYC 549 (32.7 %)
	BP-TRIG-HDL 970 (31.8 %)	COB-TRIG-GLYC 1067 (29.3 %)	INS-BP-GLYC 431 (25.7 %)
	GLYC-BP-HDL 838 (27.4 %)	COB-HDL-GLYC 926 (25.4 %)	INS-DYSL-GLYC 278 (16.5 %)
	GLYC-TRIG-WC 728 (23.8 %)		
	TRIG-HDL-WC 710 (23.2 %)		
	GLYC-HDL-WC 627 (20.5 %)		
	GLYC-TRIG-HDL 633 (20.7 %)		

*AHA/NHLBI*, American heart association/national heart, lung, and blood institute; *IDF*, International Diabetes Federation; *EGIR*, European Group for the study of Insulin Resistance; *GLYC*, hyperglycemia; *BP*, hypertension; *TRIG*, hypertriglyceridemia; *HDL*, low HDL-cholesterol; *WC*, increased waist circumference; *COB*, central obesity; *DYSL* dyslipidemia; *INS*, highest quartile of fasting insulin not having type 2 diabetes

### Prevalence of components and triads of MetS

Table 2 shows the prevalence of components and triads in each definition of MetS.

A combination of hyperglycemia, high blood pressure and central obesity was the most frequent triad within a diagnosis of MetS according to AHA/NHLBI and IDF. In the EGIR-definition, high blood pressure and central obesity together with hyperinsulinemia were most frequently prevalent in MetS-diagnosed participants.

### Risk of incident type 2 diabetes mellitus

During a median follow-up of 6.8 years 768 individuals developed type 2 diabetes mellitus. MetS was significantly associated with the risk of type 2 diabetes mellitus regardless of the definition chosen (Table 3) in cox proportional hazards models. Ethnicity did not have a significant effect and was therefore left out of the model. The cox proportional hazard ratio (HR) was 3.78 (95 % CI 3.24–4.41) for AHA/NHLBI-definition, 3.53 (95 % CI 3.01–4.14) for IDF definition, and 3.13 (95 % CI 2.69–3.64) for EGIR-definition. The risk of type 2 diabetes mellitus was highly variable dependent on the composition of diagnosis (Additional file 1: Table S2). In MetS according to AHA/NHLBI, a combination of GLYC–HDL–WC (HR 6.75; 95 % CI 5.53–8.25) was associated with the highest risk of type 2 diabetes mellitus. In MetS according to IDF, a combination of COB–HDL–GLYC (HR 6.07; 95 % CI 5.01–7.35) was associated with the highest risk of type 2 diabetes mellitus. For EGIR-diagnosis, the highest risk of type 2 diabetes mellitus was associated with a combination of INS–DYSL–GLYC (HR 7.35; 95 % CI 5.92–9.13). After correction for sex, age and individual components none of the MetS-definitions itself was significantly associated with the risk of type 2 diabetes mellitus (Table 4). The results were similar in a sensitivity analysis in which all participants with impaired fasting glucose levels (fasting glucose ≥5.6 mmol/l) were excluded from the cox regression modelling (Additional file 1: Table S7).

**Table 3:** Metabolic syndrome and hazard ratios for incident clinical endpoints

Outcome	Events in population	AHA/NHLBI	IDF	EGIR
Type 2 diabetes mellitus	765/8567	3.78 (3.24–4.41)*	3.53 (3.01–4.14)*	3.13 (2.69–3.64)*
Coronary heart disease	544/7864	1.32 (1.11–1.56)*	1.38 (1.16–1.63)*	1.08 (0.87–1.35)
Stroke	458/8304	1.29 (1.07–1.56)*	1.32 (1.10–1.59)*	0.98 (0.77–1.25)
Cardiovascular mortality	418/7724	1.21 (0.99–1.47)	1.29 (1.05–1.57)*	0.95 (0.73–1.23)
All-cause mortality	2244/8586	1.10 (1.01–1.20)*	1.09 (1.01–1.19)*	1.05 (0.94–1.17)

Data are presented as hazard ratios with 95 % confidence intervals. All analysis corrected for age and sex

*AHA/NHLBI*, American heart association/national heart, lung, and blood institute; *IDF*, International Diabetes Federation; *EGIR*, European group for the study of Insulin Resistance

* Statistically significant

**Table 4:** MetS according to different diagnosis and hazard ratios for incident clinical endpoints corrected for individual components

	AHA/NHLBI	IDF	EGIR
Type 2 diabetes mellitus	MetS 1.19 (0.90–1.58)	MetS 1.11 (0.82–1.49)	MetS 0.91 (0.56–1.49)
	GLYC 4.01 (3.30–4.87)*	GLYC 4.20 (3.45–5.12)*	GLYC 5.12 (4.38–5.98)*
	HDL 1.48 (1.24–1.76)*	HDL 1.52 (1.29–1.80)*	DYSL 1.64 (1.40–1.92)*
	WC 1.48 (1.23–1.78)*	BP 1.34 (1.08–1.65)*	WC 1.33 (1.08–1.64)*
	BP 1.28 (1.04–1.58)*	COB 1.33 (0.99–1.78)	INSUL 1.54 (0.98–2.44)
	TRIG 1.24 (1.04–1.49)*	TRIG 1.32 (1.12–1.55)*	BP 1.39 (1.18–1.64)*
Coronary heart disease	MetS 0.73 (0.53–1.01)	MetS 1.18 (0.86–1.58)	MetS 1.00 (0.55–1.81)
	BP 1.67 (1.31–2.14)*	BP 1.53 (1.20–1.95)*	BP 1.50 (1.23–1.82)*
	TRIG 1.48 (1.18–1.85)*	TRIG 1.29 (1.04–1.59)*	DYSL 1.36 (1.12–1.67)*
	WC 1.38 (1.11–1.71)*	HDL 1.17 (0.95–1.44)	WC 1.10 (0.90–1.35)
	HDL 1.34 (1.07–1.68)*	COB 0.99 (0.76–1.28)	GLYC 0.93 (0.74–1.18)
	GLYC 0.94 (0.77–1.15)	GLYC 0.82 (0.67–1.01)	INSUL 0.87 (0.50–1.52)
Stroke	MetS 1.09 (0.76–1.58)	MetS 1.12 (0.80–1.56)	MetS 0.91 (0.46–1.77)
	BP 1.44 (1.10–1.89)*	BP 1.42 (1.08–1.86)*	BP 1.44 (1.16–1.79)*
	HDL 1.36 (1.06–1.75)*	HDL 1.35 (1.07–1.71)*	GLYC 1.22 (0.96–1.56)
	WC 1.06 (0.83–1.34)	COB 1.11 (0.83–1.48)	WC 1.20 (0.95–1.52)
	GLYC 0.98 (0.78–1.22)	GLYC 0.96 (0.76–1.21)	DYSL 1.11 (0.88–1.41)
	TRIG 0.84 (0.65–1.10)	TRIG 0.84 (0.66–1.08)	INSUL 0.87 (0.47–1.61)
Cardiovascular mortality	MetS 0.86 (0.58–1.27)	MetS 1.06 (0.74–1.54)	MetS 0.79 (0.40–1.57)
	BP 1.47 (1.10–1.97)*	BP 1.39 (1.04–1.86)*	BP 1.35 (1.07–1.69)*
	TRIG 1.24 (0.94–1.62)	COB 1.21 (0.89–1.63)	WC 1.30 (1.01–1.67)*
	WC 1.21 (0.94–1.55)	TRIG 1.14 (0.89–1.47)	GLYC 1.14 (0.88–1.47)
	HDL 1.13 (0.86–1.48)	HDL 1.05 (0.81–1.35)	DYSL 1.07 (0.84–1.37)
	GLYC 1.00 (0.79–1.26)	GLYC 0.92 (0.71–1.18)	INS 1.00 (0.54–1.86)
All-cause mortality	MetS 0.97 (0.82–1.14)	MetS 0.98 (0.85–1.14)	MetS 0.81 (0.62–1.05)
	HDL 1.25 (1.11–1.41)*	HDL 1.24 (1.11–1.39)*	INSUL 1.18 (0.93–1.50)
	BP 1.09 (0.97–1.22)	BP 1.08 (0.97–1.22)	DYSL 1.15 (1.03–1.27)*
	WC 1.02 (0.92–1.13)	COB 1.05 (0.93–1.18)	GLYC 1.11 (0.99–1.24)
	GLYC 1.00 (0.90–1.10)	GLYC 0.99 (0.89–1.10)	BP 1.10 (1.00–1.21)*
	TRIG 0.97 (0.86–1.10)	TRIG 0.96 (0.86–1.08)	WC 1.04 (0.94–1.15)

Data are presented as hazard ratios with 95 % confidence intervals. All analysis corrected for age and sex

*AHA/NHLBI*, American heart association/national heart, lung, and blood institute; *IDF*, International Diabetes Federation; *EGIR*, European group for the study of Insulin Resistance; *GLYC*, hyperglycemia; *BP*, hypertension; *TRIG*, hypertriglyceridemia; *HDL*, low HDL-cholesterol; *WC*, increased waist circumference; *COB*, central obesity; *DYSL*, dyslipidemia; *INS*, highest quartile of fasting Insulin not having type 2 diabetes

* Statistically significant

### Risk of incident CHD

During a median follow-up of 7.2 years in which 544 individuals developed CHD, MetS as defined by AHA/NHLBI (HR 1.32; 95 % CI 1.11–1.56) and IDF (HR 1.38; 95 % CI 1.16–1.63 P < 0.001) were significantly associated with the risk of CHD (Table 3). In our population, the EGIR definition was not associated with the risk of incident CHD. In MetS according to AHA/NHLBI, a combination of BP–TRIG–WC (HR 1.77; 95 % CI 1.41–2.23) was associated with the highest risk of CHD (Additional file 1: Table S3). In MetS according to IDF, a combination of COB–TRIG–BP (HR 1.76; 95 % CI 1.44–2.15) was associated with the highest risk of CHD. For EGIR-diagnosis, the highest risk of CHD was associated with a combination of INS–BP–DYSL (HR 1.26; 95 % CI 0.29–1.72). After correction for age, sex and individual components none of the MetS-definitions were significantly associated with CHD (Table 4).

### Risk of incident stroke

During a median follow-up of 7.7 years in which 458 participants suffered from incident stroke, MetS according to AHA/NHLBI (HR 1.29; 95 % CI 1.07–1.56) and IDF (HR 1.32; 95 % CI 1.10–1.59) showed a significantly increased risk of stroke (Table 3). No association of the EGIR definition and incident stroke was found. In MetS according to AHA/NHLBI, a combination of GLYC–HDL–WC (HR 1.75; 95 % CI 1.31–2.34) was associated with the highest risk of stroke (Additional file 1: Table S4). In MetS according to IDF, a combination of COB–HDL–GLYC (HR 1.62; 95 % CI 1.26–2.10) was associated with the highest risk of stroke. For EGIR-diagnosis, the highest risk of stroke was associated with a combination of INS–BP–DYSL (HR 1.02; 95 % CI 0.70–1.49). After correction for age, sex and individual components none of the MetS-definitions were significantly associated with stroke (Table 4).

### Risk of cardiovascular mortality

During a median follow-up of 8.2 years in which 418 cardiovascular mortalities occurred, only the IDF-diagnosis was associated with significantly increased risk of cardiovascular mortality (HR 1.29; 95 % CI 1.05–1.57; P = 0.01) (Table 3). Within each definition, a large variability in hazard ratios for cardiovascular mortality was found (Additional file 1: Table S5). In MetS according to AHA/NHLBI, a combination of BP–TRIG–WC [HR 1.48 (95 % CI 1.13–1.94)] was associated with the highest risk of cardiovascular mortality. In MetS according to IDF, a combination of COB-TRIG-BP (HR 1.45 (95 % CI 1.13–1.85)) was associated with the highest risk of cardiovascular mortality. Neither the EGIR diagnosis nor its triads were significantly associated with cardiovascular mortality. After adjustments for age, sex and individual components none of the MetS definitions were significantly associated with cardiovascular mortality (Table 4).

### Risk of all-cause mortality

During a median follow-up of 8.7 years in which 2244 participants deceased, MetS according to AHA/NHLBI (HR 1.10 (1.01–1.20) P = 0.03) and IDF (HR 1.09 (95 % CI 1.01–1.19) P = 0.03) were associated with all-cause mortality. There was variability within definition as displayed by their triads (Additional file 1: Table S6). In MetS according to AHA/NHLBI, a combination of TRIG–HDL–WC (HR 1.24 (95 % CI 1.07–1.45)) was associated with the highest risk of all-cause mortality. In MetS according to IDF, a combination of COB–HDL–GLYC (HR 1.18 (95 % CI 1.04–1.34)) was associated with the highest risk of all-cause mortality. After adjustments for age, sex and individual components none of the diagnoses showed a significantly increased risk of all-cause mortality (Table 4).

## Discussion

In our large predominantly elderly prospective population-based study, we show there is large variability between and within the definitions of MetS with respect to prevalence- and risk estimates for important cardiovascular and metabolic clinical endpoints. In addition, we confirm that MetS does not have an additional value in the risk estimation of type 2 diabetes mellitus, CHD, stroke and mortality on top of its individual components.

MetS is a highly prevalent condition in our Dutch population. This is in line with previous reports on MetS in middle-aged and elderly populations in the United States and Europe that reported equal or higher prevalence estimates [29–31]. We diagnosed the MetS according to the definitions of AHA/NHLBI, IDF and EGIR. The IDF-definition diagnosed the largest proportion of our population with MetS, followed by AHA/NHLBI and EGIR respectively, which is similar to previous studies [5, 32, 33]. This can be explained by the lower IDF cut-off points for waist circumference and BMI, resulting in more individuals that meet the central obesity-criterium. The EGIR-diagnosis selects an upper quartile of fasting insulin and excludes prevalent diabetes, resulting in a lower prevalence compared to the other definitions.

In our population, MetS is a strong risk factor for type 2 diabetes mellitus regardless of the definition chosen. This has already been found by several study groups in predominantly middle aged populations of various ethnicities [12, 15, 34, 35]. Sattar et al. also confirmed this association in elderly, predominantly male subjects and subjects at risk for cardiovascular disease [19]. However, these studies were partly based on self-reported data and the associations were mostly the result of the hyperglycemic component rather than the diagnosis of MetS itself. Our findings are in line with this study, since the association of MetS with type 2 diabetes mellitus disappears after correcting for its components of which the hyperglycemic component constitutes the largest hazard. Our study, being population-based and with larger and meticulous follow-up, therefore adds to the evidence provided by previous studies that MetS does not confer additional risk of type 2 diabetes mellitus above the sum of its components, especially fasting glucose [15, 19].

MetS is a known risk factor for CVD in middle aged and elderly populations [13, 14, 19, 36]. We found a relatively weak association of MetS with CVD in concordance with previous associations reported in literature [19]. Our study adds to previous studies including a large meta-analysis [14] that show that MetS does not show additive value to the risk associated with the sum of its individual components [1, 2, 4, 5, 36]. Previous studies did find an independent associative role of MetS [37] and higher hazard ratios for MetS and incident cardiovascular events [38]. However, these studies were done in small numbers of patients at younger age having essential hypertension [37] or being suspected of having coronary artery disease [38]. Therefore, those results may not be similar to our study, which is a population-based study with predominantly elderly participants. For stroke in particular, Kotani et al. found MetS to have a positive association with stroke in women in a retrospective cohort [39]. We found MetS to be associated with stroke in the general population, but the association disappeared after correcting for the individual MetS components.

Although earlier studies on middle-aged younger individuals suggested otherwise [11, 13, 14, 40], we did not find any significant associations of MetS with all-cause mortality after correction for its individual components in any of the definitions. This could very well be an effect of the relatively higher age of our population making study subjects equally prone to decease due to causes other than cardiometabolic disease, thereby reducing the relative effect of MetS. Our findings on all-cause mortality are in line with results obtained from patients after coronary artery bypass grafting (CABG) in which survival of MetS patients without diabetes resembled their matched background population [41].

Remarkably dyslipidemia and blood pressure were the main contributing factors for cardiovascular disease and cardiovascular- and all-cause mortality effects of MetS. Although these are known as important independent risk factors for coronary heart disease and atherosclerosis [42–47], this finding adds to the evidence that these individual components important predictors in CVD [19].

The strengths of this study are the large sample size, population-based design and the long-term follow-up. Furthermore, data extraction has been done in a systematic way.

Despite the fact that we have executed this study with great care, we have to address some limitations of our study. Participants included in the Rotterdam study were mainly European Caucasians (97.8 %). Therefore our results may not apply to other ethnic groups. Considering the dynamic changes in European demographic, our results should be interpreted accordingly. Unfortunately a small proportion (1.7 %) of our population had missing data for the definition of MetS. We addressed this by applying a reliable multiple imputation method.

In this study, we approach the MetS as a predictive tool to identify patients at high risk for cardiometabolic endpoints. However as Tenenbaum and Fisman emphasized [48], MetS is still an interesting biological feature of coexistence of components. Research directed at the underlying mechanisms of their coexistence could lead to important biological insights in underlying cardiometabolic disease pathophysiology. These studies are beyond the scope of our current epidemiological approach for prediction purposes.

In conclusion, MetS shows high variability in its association with clinical endpoints both within and between diagnoses according to different definitions. Also, in a relatively old population MetS did not have additional predictive value on top of its components for any of the cardiometabolic endpoints. Besides as a manner of easy identification of risk patients, MetS does not seem to add any predictive value for clinical practice.

## Additional file

10.1186/s12933-016-0387-4 Overview of definitions of MetS. **Table S2.** The prevalence of triads of MetS and risk of incident T2D. **Table S3.** The prevalence of triads of MetS and risk of incident CHD. **Table S4.** The prevalence of triads of MetS and risk of incident stroke. **Table S5.** The prevalence of triads of MetS and risk of cardiovascular mortality. **Table S6.** The prevalence of triads of MetS and risk of all-cause mortality. **Table S7.** Univariate and multivariate analysis of metabolic syndrome and hazard ratios for incident type 2 diabetes mellitus (excluding participants with impaired fasting glucose levels).

## Abbreviations

AHA/NHLBI: American Heart Association/National Heart, Lung and Blood Institute
BP: blood pressure component
COB: central obesity component
CVD: cardiovascular disease
DYSL: dyslipidemia component
EGIR: European Group for the Study of Insulin Resistance
GLYC: hyperglycemia component
HDL: low HDL-cholesterol component
IDF: International Diabetes Federation
INS: hyperinsulinemia component
MetS: metabolic Syndrome
TRIG: triglyceride component
WC: waist circumference
WHO: World Health Organization
