# Supplementary material for: The clinical value of metabolic syndrome and risks of cardiometabolic events and mortality in the elderly: the Rotterdam study
Source: Cardiovasc Diabetol. 2016 Apr 27;15:69. doi: 10.1186/s12933-016-0387-4 (PMC4847340; doi:10.1186/s12933-016-0387-4)
Supplement: Supplementary file 1 — 10.1186/s12933-016-0387-4 Overview of definitions of MetS. Table S2. The prevalence of triads of MetS and risk of incident T2D. Table S3. The prevalence of triads of MetS and risk of incident CHD. Table S4. The prevalence of triads of MetS and risk of incident stroke. Table S5. The prevalence of triads of MetS and risk of cardiovascular mortality. Table S6. The prevalence of triads of MetS and risk of all-cause mortality. Table S7. Univariate and multivariate analysis of metabolic syndrome and hazard ratios for incident type 2 diabetes mellitus (excluding participants with impaired fasting glucose levels). [file 12933_2016_387_MOESM1_ESM.doc]

Supplemental table S1: Overview of definitions of MetS

|  | AHA/NHLBI (Grundy 2005) | IDF  (Alberti 2005) | EGIR  (Balkau 1999) | Harmonizing  (Alberti, Grundy 2009) |
| --- | --- | --- | --- | --- |
| GLYC- Component | ≥ 5.6 mmol/L  Or treatment | ≥ 5.6 mmol/L Or type 2 diabetes | ≥ 6.1 mmol/L | ≥ 5.6 mmol/L  Or treatment |
| HDL-C  Component | Men < 1.03 Women < 1.29 Or treatment | Men < 1.03 Women < 1.29  Or treatment | *-* | Men < 1.03 Women < 1.29  Or treatment |
| TG-  Component | Fasting  ≥1.7 mmol/L  Or treatment | Fasting  ≥1.7 mmol/L  Or treatment | *-* | Fasting  ≥1.7 mmol/L  Or treatment |
| Dyslipidemia  Component | - | - | TG >2.0mmol/L OR  HDL<1.0mmol/L  OR Treatment | - |
| Obesity component | Men ≥ 102cm  Women ≥ 88cm | Men ≥ 94 cm  Women ≥ 80 cm  Or BMI > 30kg/m2 | Men ≥ 94 cm  Women ≥ 80 cm | Men ≥ 102cm  Women ≥ 88cm |
| Blood pressure  component | SBP ≥ 130mmHg  DBP ≥ 85mmHg  Or treatment | SBP ≥ 130mmHg  DBP ≥ 85mmHg  Or treatment | SBP ≥ 140mmHg  DBP ≥ 90 mmHg  Or treatment | SBP ≥ 130mmHg  DBP ≥ 85mmHg  Or treatment |

GLYC, glucose; HDL-C, HDL-cholesterol; TG, triglycerides; TG, triglycerides; SBP systolic blood pressure; DBP, diastolic blood pressure

Supplemental table S2: The prevalence of triads of MetS and risk of incident T2D.

| AHA/NHLBI | | IDF | | EGIR | |
| --- | --- | --- | --- | --- | --- |
| Triads | HR | *Triads* | *HR* | *Triads* | *HR* |
| GLYC-HDL-WC | 6.75 (5.53-8.25)^*^ | COB-HDL-GLYC | 6.07 (5.01-7.35)^*^ | INS-DYSL-GLYC | 7.35 (5.92-9.13)^*^ |
| GLYC-TRIG-HDL | 5.98 (4.90-7.30)^*^ | COB-TRIG-GLYC | 5.44 (4.52-6.55)^*^ | INS-BP-GLYC | 6.72 (5.59-8.09)^*^ |
| GLYC-BP-HDL | 5.88 (4.88-7.09)^*^ | COB-GLYC-BP | 4.57 (3.88-5.39)^*^ | INS-WC-GLYC | 6.17 (5.20-7.32)^*^ |
| GLYC-TRIG-WC | 5.70 (4.70-6.92)^*^ | COB-TRIG-HDL | 4.24 (3.47-5.18)^*^ | INS-BP-DYSL | 4.19 (3.46-5.08)^*^ |
| GLYC-BP-TRIG | 5.17 (4.32-6.19)^*^ | COB-HDL-BP | 4.03 (3.34-4.86)^*^ | INS-WC-DYSL | 3.77 (3.14-4.52)^*^ |
| GLYC-BP-WC | 5.05 (4.27-5.98)^*^ | COB-TRIG-BP | 3.84 (3.20-4.61)^*^ | INS-WC-BP | 3.21 (2.73-3.76)^*^ |
| BP-TRIG-HDL | 4.17 (3.43-5.07)^*^ |  | | | |
| BP-HDL-WC | 4.43 (3.62-5.42)^*^ |
| TRIG-HDL-WC | 4.48 (3.60-5.58)^*^ |
| BP-TRIG-WC | 4.05 (3.34-4.91)^*^ |

Data are presented as hazard ratios with 95% confidence intervals. All analysis corrected for age and sex. ^*^ = statistically significant. GLYC, hyperglycemia; BP, hypertension; TRIG, hypertriglyceridemia; HDL, low HDL-cholesterol; WC, increased waist circumference; COB, central obesity; DYSL dyslipidemia; INS, highest quartile of fasting Insulin not having type 2 diabetes.

Supplemental table S3: The prevalence of triads of MetS and risk of incident CHD.

| AHA/NHLBI | | IDF | | EGIR | |
| --- | --- | --- | --- | --- | --- |
| Triads | HR | Triads | HR | Triads | HR |
| BP-TRIG-WC | 1.77 (1.41-2.23)^*^ | COB-TRIG-BP | 1.76 (1.44-2.15)^*^ | INS-BP-DYSL | 1.26 (0.92-1.72) |
| TRIG-HDL-WC | 1.71 (1.28-2.27)^*^ | COB-TRIG-HDL | 1.55 (1.21-1.99)^*^ | INS-WC-BP | 1.19 (0.94-1.50) |
| GLYC-TRIG-WC | 1.61 (1.24-2.11)^*^ | COB-TRIG-GLYC | 1.52 (1.20-1.93)^*^ | INS-WC-DYSL | 1.13 (0.84-1.51) |
| BP-HDL-WC | 1.58 (1.23-2.04)^*^ | COB-HDL-BP | 1.47 (1.18-1.84)^*^ | INS-BP-GLYC | 1.07 (0.73-1.55) |
| BP-TRIG-HDL | 1.48 (1.16-1.90)^*^ | COB-HDL-GLYC | 1.34 (1.03-1.75)^*^ | INS-DYSL-GLYC | 1.06 (0.67-1.68) |
| GLYC-TRIG-HDL | 1.43 (1.06-1.92)^*^ | COB-GLYC-BP | 1.26 (1.04-1.53)^*^ | INS-WC-GLYC | 0.90 (0.63-1.29) |
| GLYC-HDL-WC | 1.42 (1.04-1.93)^*^ |  | | | |
| GLYC-BP-TRIG | 1.39 (1.10-1.77)^*^ |
| GLYC-BP-WC | 1.31 (1.05-1.62)^*^ |
| GLYC-BP-HDL | 1.28 (0.98-1.66) |

Data are presented as hazard ratios with 95% confidence intervals. All analysis corrected for age and sex. ^*^ = statistically significant. GLYC, hyperglycemia; BP, hypertension; TRIG, hypertriglyceridemia; HDL, low HDL-cholesterol; WC, increased waist circumference; COB, central obesity; DYSL dyslipidemia; INS, highest quartile of fasting Insulin not having type 2 diabetes. Corrected for age and sex. ^*^= statistically significant

Supplemental table S4: The prevalence of triads of MetS and risk of incident stroke.

| AHA/NHLBI | | IDF | | EGIR | |
| --- | --- | --- | --- | --- | --- |
| Triads | HR | Triads | HR | Triads | HR |
| GLYC-HDL-WC | 1.75(1.31-2.34)^*^ | COB-HDL-GLYC | 1.62 (1.26-2.10)^*^ | INS-BP-DYSL | 1.02 (0.70-1.49) |
| BP-HDL-WC | 1.62 (1.24-2.10)^*^ | COB-HDL-BP | 1.55 (1.24-1.96)^*^ | INS-BP-GLYC | 1.10 (0.75-1.63) |
| TRIG-HDL-WC | 1.59 (1.17-2.16)^*^ | COB-TRIG-HDL | 1.34 (1.01-1.77)^*^ | INS-WC-GLYC | 1.06 (0.74-1.51) |
| GLYC-BP-HDL | 1.51 (1.17-1.97)^*^ | COB-GLYC-BP | 1.28 (1.04-1.57) | INS-WC-BP | 1.04 (0.80-1.36) |
| GLYC-TRIG-WC | 1.39 (1.03-1.88)^*^ | COB-TRIG-BP | 1.28 (1.00-1.63)^*^ | INS-WC-DYSL | 1.00 (0.71-1.41) |
| GLYC-TRIG-HDL | 1.39 (1.01-1.90)^*^ | COB-TRIG-GLYC | 1.25 (0.95-1.65) | INS-DYSL-GLYC | 0.73 (0.40-1.33) |
| BP-TRIG-WC | 1.37 (1.05-1.79)^*^ |  | | | |
| GLYC-BP-WC | 1.31 (1.04-1.64)^*^ |
| BP-TRIG-HDL | 1.30 (0.99-1.73) |
| GLYC-BP-TRIG | 1.22 (0.92-1.60) |

Data are presented as hazard ratios with 95% confidence intervals. All analysis corrected for age and sex. ^*^ = statistically significant. GLYC, hyperglycemia; BP, hypertension; TRIG, hypertriglyceridemia; HDL, low HDL-cholesterol; WC, increased waist circumference; COB, central obesity; DYSL dyslipidemia; INS, highest quartile of fasting Insulin not having type 2 diabetes.

Supplemental table S5: The prevalence of triads of MetS and risk of cardiovascular mortality.

| AHA/NHLBI | | IDF | | EGIR | |
| --- | --- | --- | --- | --- | --- |
| Triads | HR | Triads | HR | Triads | HR |
| BP-TRIG-WC | 1.48 (1.13-1.94)^*^ | COB-TRIG-BP | 1.45 (1.13-1.85)^*^ | INS-WC-BP | 1.02 (0.77-1.33) |
| GLYC-TRIG-WC | 1.44 (1.06-1.95)^*^ | COB-TRIG-GLYC | 1.44 (1.09-1.90)^*^ | INS-BP-DYSL | 0.96 (0.64-1.44) |
| GLYC-BP-TRIG | 1.37 (1.05-1.81)^*^ | COB-GLYC-BP | 1.28 (1.03-1.59)^*^ | INS-WC-DYSL | 0.91 (0.62-1.34) |
| BP-TRIG-HDL | 1.25 (0.93-1.67) | COB-HDL-GLYC | 1.25 (0.93-1.67) | INS-BP-GLYC | 0.89 (0.57-1.39) |
| TRIG-HDL-WC | 1.33 (0.94-1.87) | COB-HDL-BP | 1.26 (0.97-1.62) | INS-WC-GLYC | 0.87 (0.57-1.31) |
| GLYC-TRIG-HDL | 1.31 (0.94-1.83) | COB-TRIG-HDL | 1.30 (0.97-1.76) | INS-DYSL-GLYC | 0.68 (0.35-1.33) |
| GLYC-BP-HDL | 1.20 (0.90-1.59) |  | | | |
| BP-HDL-WC | 1.18 (0.88-1.59) |
| GLYC-BP-WC | 1.18 (0.93-1.50) |
| GLYC-HDL-WC | 1.16 (0.82-1.63) |

Data are presented as hazard ratios with 95% confidence intervals. All analysis corrected for age and sex. ^*^ = statistically significant. GLYC, hyperglycemia; BP, hypertension; TRIG, hypertriglyceridemia; HDL, low HDL-cholesterol; WC, increased waist circumference; COB, central obesity; DYSL dyslipidemia; INS, highest quartile of fasting Insulin not having type 2 diabetes.

Supplemental table S6: The prevalence of triads of MetS and risk of all-cause mortality.

| AHA/NHLBI | | IDF | | EGIR | |
| --- | --- | --- | --- | --- | --- |
| Triads | HR | Triads | HR | Triads | HR |
| TRIG-HDL-WC | 1.24 (1.07-1.45)^*^ | COB-HDL-GLYC | 1.18 (1.04-1.34)^*^ | INS-BP-GLYC | 1.07 (0.89-1.29) |
| GLYC-TRIG-HDL | 1.24 (1.07-1.44)^*^ | COB-HDL-BP | 1.17 (1.05-1.31)^*^ | INS-WC-BP | 1.05 (0.93-1.19) |
| GLC-HDL-WC | 1.20 (1.04-1.39)^*^ | COB-TRIG-HDL | 1.18 (1.04-1.33)^*^ | INS-WC-DYSL | 1.03 (0.88-1.20) |
| GLYC-TRIG-WC | 1.11 (0.96-1.28) | COB-TRIG-GLYC | 1.06 (0.93-1.20) | INS-WC-GLYC | 1.02 (0.86-1.21) |
| BP-HDL-WC | 1.20 (1.05-1.37)^*^ | COB-TRIG-BP | 1.08 (0.97-1.21) | INS-BP-DYSL | 1.02 (0.86-1.20) |
| BP-TRIG-HDL | 1.17 (1.03-1.33)^*^ | COB-GLYC-BP | 1.06 (0.96-1.16) | INS-DYSL-GLYC | 1.00 (0.79-1.28) |
| GLYC-BP-HDL | 1.16 (1.03-1.32)^*^ |  | | | |
| BP-TRIG-WC | 1.14 (1.01-1.30)^*^ |
| GLYC-BP-WC | 1.05 (0.94-1.17) |
| GLYC-BP-TRIG | 1.05 (0.92-1.19) |

Data are presented as hazard ratios with 95% confidence intervals. All analysis corrected for age and sex. ^*^ = statistically significant. GLYC, hyperglycemia; BP, hypertension; TRIG, hypertriglyceridemia; HDL, low HDL-cholesterol; WC, increased waist circumference; COB, central obesity; DYSL dyslipidemia; INS, highest quartile of fasting Insulin not having type 2 diabetes.

Supplemental table S7: Univariate and multivariate analysis of metabolic syndrome and hazard ratios for incident type 2 diabetes mellitus (excluding participants with impaired fasting glucose levels).

|  | Events in population | AHA/NHLBI | IDF | EGIR |
| --- | --- | --- | --- | --- |
| Type 2 diabetes mellitus | 174/5167 | MetS 2.41 (1.75-3.31)^*^ | MetS 2.81 (1.49-2.81)^*^ | MetS 2.81 (1.93-4.07)^*^ |
|  |  | MetS 1.21 (0.65-2.26)  TRIG 1.42 (0.92-2.18) HDL 1.40 (0.91-2.15)  WC 1.36 (0.95-1.95) BP 1.34 (0.91-1.96) | MetS 0.88 (0.50-1.57)  TRIG 1.63 (1.06-2.50)^*^ HDL 1.58 (1.05-2.39)^*^  COB 1.44 (0.95-2.17) BP 1.44 (0.98-2.11) | MetS 0.85 (0.39-1.86)  DYSL 2.18 (1.54-3.07)^*^  INSUL 2.14 (1.08-4.24)^*^  BP 1.48 (1.05-2.09)^*^  WC 1.21 (0.83-1.77) |

Data are presented as hazard ratios with 95% confidence intervals. All analysis corrected for age and sex. ^*^ = statistically significant. AHA/NHLBI, American heart association / national heart, lung, and blood institute; IDF, International Diabetes Federation; EGIR, European group for the study of Insulin Resistance.
